# Analysis of the role of PANoptosis in seizures via integrated bioinformatics analysis and experimental validation

**DOI:** 10.1016/j.heliyon.2024.e26219

**Published:** 2024-02-14

**Authors:** Yueying Liu, Yuanjin Chang, Xiaofan Jiang, Huiya Mei, Yingsi Cao, Dongqin Wu, Ruijin Xie, Wenjun Jiang, Emely Vasquez, Yu Wu, Shunyan Lin, Yachuan Cao

**Affiliations:** a Affiliated Hospital of Jiangnan University, Wuxi, China; b Wuxi School of Medicine, Jiangnan University, Wuxi, China; c Clinical Medical College, Yangzhou University, Yangzhou, China; d The City University of New York School of Medicine, New York, USA

**Keywords:** PANoptosis, Epilepsy, Cell death, Bioinformatics

## Abstract

**Background:**

Epilepsy is recognized as the most common chronic neurological condition among children, and hippocampal neuronal cell death has been identified as a crucial factor in the pathophysiological processes underlying seizures. In recent studies, PANoptosis, a newly characterized form of cell death, has emerged as a significant contributor to the development of various neurological disorders, including Alzheimer's disease, Parkinson's disease, and amyotrophic lateral sclerosis. PANoptosis involves the simultaneous activation of pyroptosis, apoptosis, and necroptosis within the same population of cells. However, its specific role in the context of seizures remains to be fully elucidated. Further investigation is required to uncover the precise involvement of PANoptosis in the pathogenesis of seizures and to better understand its potential implications for the development of targeted therapeutic approaches in epilepsy.

**Methods:**

In this study, the gene expression data of the hippocampus following the administration of kainic acid (KA) or NaCl was obtained from the Gene Expression Omnibus (GEO) database. The PANoptosis-related gene set was compiled from the GeneCards database and previous literature. Time series analysis was performed to analyze the temporal expression patterns of the PANoptosis-related genes. Gene set variation analysis (GSVA), Gene ontology (GO), and Kyoto encyclopedia of genes and genomes (KEGG) were employed to explore potential biological mechanisms underlying PANoptosis and its role in seizures. Weighted gene co-expression network analysis (WGCNA) and differential expression analysis were utilized to identify pivotal gene modules and PANoptosis-related genes associated with the pathophysiological processes underlying seizures. To validate the expression of PANoptosis-related genes, Western blotting or quantitative real-time polymerase chain reaction (qRT-PCR) assays were conducted. These experimental validations were performed in human blood samples, animal models, and cell models to verify the expression patterns of the PANoptosis-related genes and their relevance to epilepsy.

**Results:**

The GSVA analysis performed in this study demonstrated that PANoptosis-related genes have the potential to distinguish between the control group and KA-induced epileptic mice. This suggests that the expression patterns of these genes are significantly altered in response to KA-induced epilepsy. Furthermore, the Weighted gene co-expression network analysis (WGCNA) identified the blue module as being highly associated with epileptic phenotypes. This module consists of genes that exhibit correlated expression patterns specifically related to epilepsy. Within the blue module, 10 genes were further identified as biomarker genes for epilepsy. These genes include MLKL, IRF1, RIPK1, GSDMD, CASP1, CASP8, ZBP1, CASP6, PYCARD, and IL18. These genes likely play critical roles in the pathophysiology of epilepsy and could serve as potential biomarkers for diagnosing or monitoring the condition.

**Conclusion:**

In conclusion, our study suggests that the hippocampal neuronal cell death in epilepsy may be closely related to PANoptosis, a novel form of cell death, which provides insights into the underlying pathophysiological processes of epilepsy and helps the development of novel therapeutic approaches for epilepsy.

## 1 Introduction

Epilepsy is a neurological condition characterized by recurrent, unprovoked seizures resulting from sudden surges of abnormal electrical activity in the brain [1]. Notably, it affects approximately 0.5%–1% of children globally, making it one of the most common neurological disorders in children, with the highest incidence occurring within the first year of life [2]. Antiepileptic drugs (AEDs) represent the first-line treatment for epilepsy, and with appropriate management, many children with epilepsy can lead normal and fulfilling lives [3]. On one hand, AEDs can induce side effects in children, including dizziness, nausea, vomiting, depression, irritability, and cognitive dysfunction. On the other hand, about 20%–30% of children with epilepsy do not respond well to AEDs and continue to experience seizures even with adequate treatment [4]. Consequently, there is a pressing need for novel therapeutic strategies to address these issues.

The etiology of epilepsy is multifaceted, and the exact pathophysiological mechanism underlying the condition remains elusive. Neuronal cell death is widely accepted as one of the primary causes of epilepsy, with clinical and pathological evidence suggesting that hippocampal neuronal cell death is a significant feature of the disorder [5]. Moreover, hippocampal neuronal cell death can impair cognitive functions and elevate the risk of complications in epilepsy. However, the precise mechanisms underlying hippocampal neuron cell death in epilepsy are still not fully understood. Therefore, it is important to investigate the molecular mechanism of hippocampal neuron cell death in epilepsy.

Programmed cell death (PCD) is a form of cell death where the cell contributes actively to its own demise [6]. It is a crucial process that occurs in the nervous system, known as neuronal cell death, and plays a critical role in various neurological diseases, including amyotrophic lateral sclerosis, Alzheimer's disease, Parkinson's disease, and epilepsy [6]. Several types of PCD, including apoptosis, autophagy, ferroptosis, pyroptosis, and necroptosis, are observed in animal models of epilepsy and epileptic states (SE) [[7], [8], [9]]. Targeting PCD pathways may offer novel therapeutic opportunities for managing epilepsy. Recently, a new form of inflammatory-programmed cell death, termed PANoptosis, has been identified. PANoptosis involves complex coordination and crosstalk between pyroptosis, apoptosis, and necroptosis, regulated by multifaceted PANoptosome complexes [10]. Neuroinflammation has been recognized as a crucial factor in the development and progression of several neurological diseases [11]. Furthermore, emerging evidence suggests that PANoptosis may play a role in neurological disorders beyond cancer and infectious diseases, such as ischemic stroke and Alzheimer's disease [10,12,13]. However, the investigation into the potential involvement of PANoptosis in epilepsy remains limited.

Hence, the aim of this study was to explore the involvement of PANoptosis in epilepsy using an integrated approach of bioinformatics analysis and experimental validation. The outcomes of this investigation will not only enhance our comprehension of the molecular mechanisms implicated in seizures but also have the potential to reveal novel therapeutic targets for the treatment of epilepsy.

## 2 Materials and methods

### 2.1 Data download and preprocessing

**Fig. 1:**
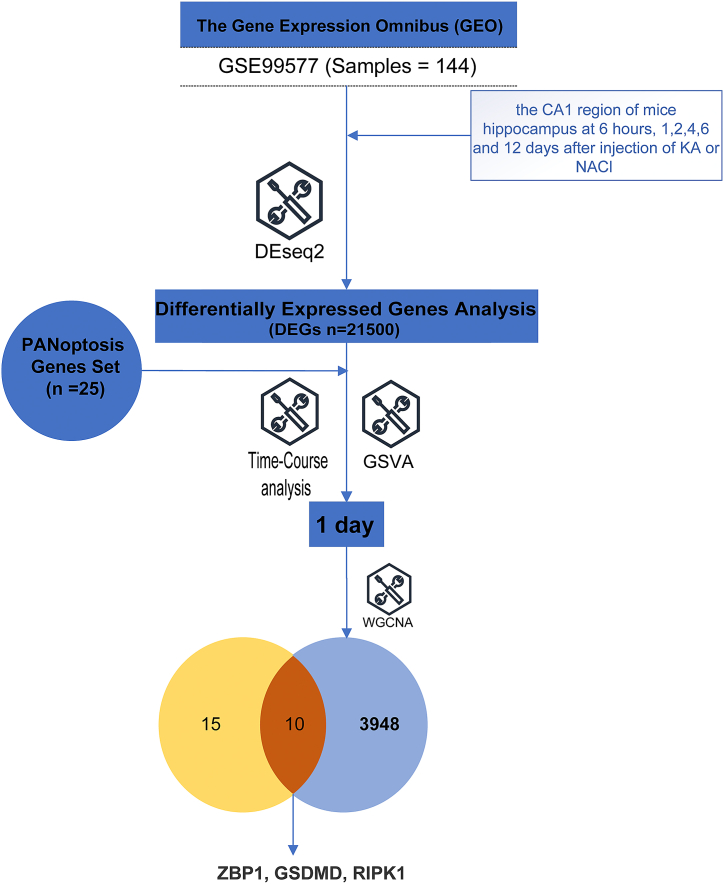
The graphic abstract of this research.

The datasets used in this study were obtained from the Gene Expression Omnibus (GEO) database (http://www.ncbi.nlm.nih.gov/geo/), specifically dataset GSE99577. This dataset comprises gene expression data in the CA1 region of the mouse hippocampus at specific time intervals: 6 h, 1 day, 2 days, 4 days, 6 days, and 12 days following the administration of kainic acid (KA, the KA group) or NaCl (the control group). To facilitate subsequent analysis, the raw gene counts were normalized to per million transcripts (TPM). A graphical abstract illustrating the process of this research is presented in Fig. 1A.

### 2.2 Acquisition of PANoptosis-related genes

**Fig. 2:**
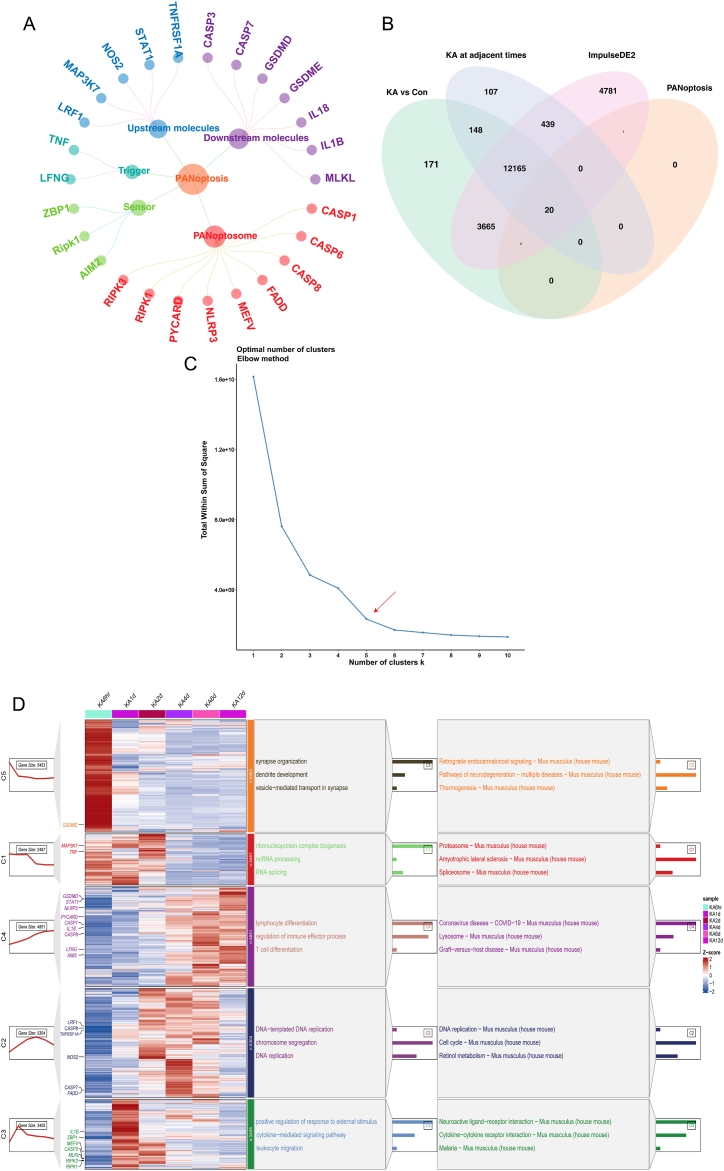
Analysis of time-course expression patterns of PANoptosis gene set. (A) Component of PANoptosis. Light green represents PANoptosis-sensor; turquoise represents PANoptosis-trigger; light red represents PANoptosome; light blue represents upstream molecules of PANoptosis; and light purple represents downstream molecules of PANoptosis. (B) The number of differentially expressed genes (DEGs). (C) Optimal number of clusters. The optimal number of clusters (K) was set to 5 since K beyond 5 starts to exhibit diminishing improvement of the sum of squares. (D) Time-course transcriptome analysis. Middle: heatmap of gene expression in different clusters. Line graph on the left: the median line of different cluster expression patterns. Right: GO enrichment analysis and KEGG enrichment analysis results of different clusters. Select and display the top 5 items individually according to -log 10(P-value) ordering. (For interpretation of the references to colour in this figure legend, the reader is referred to the Web version of this article.)

In this study, a total of 26 PANoptosis-related genes were obtained from a combination of the GeneCards database (https://www.genecards.org/) and previous literature [[14], [15], [16], [17], [18], [19], [20], [21], [22]]. Detailed information about each of the genes investigated in this study can be found in Table 1. Additionally, the components of PANoptosis are illustrated in Fig. 2A.

**Table 1:** Detailed information of PANoptosis genes.

Component of PANoptosis	Key gene	Reference
Upstream molecules	STAT1	Rajendra Karki et al., 2021 [14]
Upstream molecules	INOS	Rajendra Karki et al., 2021 [14]
Upstream molecules	MAP3K7(TAK1)	R. K. Subbarao Malireddi et al., 2019 [15]
Upstream molecules	TNFR1(Tnfrsf1a)	Rajendra Karki et al., 2021 [15]
Upstream molecules	IRF1	Teneema Kuriakose et al., 2016 [16]
Trigger	TNF	Genecards database
Trigger	IFNG(IFNs)	Rajendra Karki et al., 2021 [15]
Sensor	ZBP1(DAI/DLM1)	Teneema Kuriakose et al., 2016 [16]
Sensor	AIM2	Genecards database
Sensor	RIPK1	Genecards database
PANoptosome	NLRP3	Teneema Kuriakose et al., 2016 [16]
PANoptosome	CASP8	Shelbi Christgen et al., 2020 [17]
PANoptosome	FADD	Prajwal Gurung et al., 2014 [18]
PANoptosome	CASP1	Yaqiu Wang., 2021 [19]
PANoptosome	PYCARD (ASC)	SangJoon Lee et al., 2021 [20]
PANoptosome	RIPK1	R. K. Subbarao Malireddi et al., 2019 [15]
PANoptosome	MEFV(Pyrin)	Genecards database
PANoptosome	CASP6	Min Zheng et al., 2020 [21]
PANoptosome	RIPK3	Min Zheng et al., 2020 [21]
Downstream molecules	IL18	Genecards database
Downstream molecules	IL1B	Genecards database
Downstream molecules	CASP3	Lorenzo Galluzzi et al., 2018 [22]
Downstream molecules	CASP7	Lorenzo Galluzzi et al., 2018 [22]
Downstream molecules	GSDMD	Genecards database
Downstream molecules	GSDME(DFNA5)	Genecards database
Downstream molecules	MLKL	Genecards database

### 2.3 Identification of differentially expressed genes (DEGs) analysis

DEGs were conducted on the TPM normalized data from GSE99577 using the DESeq2 package in R (version 4.2.2) following the methodology described in a previous study [23]. The Wald test was employed to generate p-values and log2 fold changes. Genes meeting the criteria of adjusted P-values <0.05 and absolute log2 fold changes (the KA group/the control group) > 1 were classified as DEGs.

### 2.4 Time-course transcriptome analysis

In order to conduct a time-series differential expression analysis between the KA group and the control group at different time points, the ImpulseDE2 package in R was employed. The analysis was performed following the methodology outlined in a previous study [24]. Raw counts were used as input data for the analysis.

### 2.5 Soft clustering analysis

In this study, the Mfuzz package, which allows for soft clustering analysis by assigning genes to multiple clusters based on their clustering coefficient, was utilized. The package was employed to perform soft clustering analysis on the differentially expressed genes (DEGs). Subsequently, the R package ClusterGVis was used to visualize the results of the clustering analysis [25].

### 2.6 Gene ontology (GO) annotation analysis

Gene Ontology (GO) annotation analysis was carried out using the "clusterProfiler" R package in this study. A significance threshold of p-value <0.05 was set to determine statistically significant results in the analysis [26].

### 2.7 Kyoto encyclopedia of genes and genomes (KEGG) annotation analysis

In this study, Kyoto Encyclopedia of Genes and Genomes (KEGG) Annotation Analysis was performed using the "clusterProfiler" R package. A statistical significance threshold of p-value <0.05 was set to determine significant results in the analysis [26].

### 2.8 Gene set variation analysis (GSVA)

In this study, GSVA was conducted using the "GSVA" package (version 1.46.0) in R [27]. The background gene sets for GSVA were defined using the PANoptosis-related genes. To analyze the differences in the GSVA scores among all samples, the "limma" package (version 3.54.2) was employed. The screening criteria for identifying significant differences were set as |t| > 2 and p < 0.05.

### 2.9 Weighted gene co-dxpression network analysis (WGCNA)

To identify gene co-expression modules, Weighted gene co-expression network analysis (WGCNA) was performed on the transcriptome data from the 1-day time point using the WGCNA package (version 1.72.1) in R Studio [28]. The TPM values of gene expression were used as input for the WGCNA package to construct gene networks. A signed weighted correlation matrix, consisting of pairwise Pearson correlation coefficients, was computed using a soft thresholding parameter (β) set to 12, which helped achieve a scale-free topology model. Based on this adjacency matrix, the topological overlap measure, which quantifies the interconnectedness of the network, was calculated. Hierarchical clustering with average linkage was then employed to group highly correlated genes together. The WGCNA dynamic hybrid tree-cut algorithm was utilized to identify co-expression network modules. The detection of network modules involved applying a dynamic tree-cut algorithm with a minimum cluster size of 30 and a merging threshold of 0.25. Hub genes were identified based on their eigengene connectivity (KME), which indicates the strength of their association with the module.

### 2.10 Identification of PANoptosis-related diagnostic biomarkers for epilepsy

In this study, the diagnostic performance of the PANoptosis-related biomarkers was assessed using the receiver operating characteristic (ROC) curve and area under the curve (AUC) [29]. First, the ROC curve was generated to evaluate the discrimination ability of the biomarkers. The ROC curve plots the actual positive rate (sensitivity) against the false positive rate (1 - specificity) at different classification thresholds. The AUC, which represents the overall performance of the biomarkers, was calculated by measuring the area under the ROC curve. A higher AUC indicates better diagnostic accuracy. Additionally, a logistic regression model was constructed based on 10 selected marker genes. The glm function in the R package was used to build the logistic regression model, and the predict function was employed to predict the sample types in the GSE99577 dataset. The diagnostic power of the logistic regression model was assessed using ROC curves, similar to the evaluation of the individual gene biomarkers.

### 2.11 Single-gene gene set enrichment analysis (ssGSEA)

The GSEA (version 1.2) package in R Studio was employed for this analysis. The TPM values of gene expression were used as input for the ssGSEA package to explore the related pathways associated with the PANoptosis-related diagnostic biomarkers [30]. To identify the pathways relevant to the diagnostic biomarkers, the correlation between the PANoptosis-related diagnostic biomarkers and all other genes in the 1-day transcriptome data of the GSE99577 dataset was calculated. The genes were then sorted in descending order based on their correlations. This sorted gene list was considered as the gene set to be tested for pathway enrichment analysis. The analysis further involved the utilization of the predefined KEGG signaling pathway set to evaluate the enrichment of the sorted genes in the KEGG pathways. This step aimed to identify the specific KEGG pathways that demonstrated significant enrichment among the genes associated with the PANoptosis-related diagnostic biomarkers.

### 2.12 Human blood samples

To investigate the differences in ZBP1, GSDMD, and RIPK1 expression between patients with seizures and healthy children, a pilot study was conducted at the Pediatrics Department of the Affiliated Hospital of Jiangnan University. The study collected blood samples from 5 healthy school-aged children and age-matched, newly diagnosed, untreated patients with seizures. The clinical characteristics of the patients with seizures are summarized in Table 2. Total RNA was extracted from leukocytes using the PAXgene Blood RNA Kit (762174, QIAGEN Co., Ltd, Hilden, Germany), following the methodology described in our previous work [9]. Quantitative real-time reverse transcription PCR (qRT-PCR) was performed to assess the mRNA expression levels of ZBP1. The primers used in this pilot study are shown in Table 4. This pilot study received approval from the Research Ethics Committees of the Affiliated Hospital of Jiangnan University (Approval number:2023JNky-231), ensuring adherence to ethical guidelines and protecting the rights and well-being of the study participants.

**Table 2:** The clinical characteristics of the patients with seizures.

Clinical characteristics	
Age (years, mean ± SD)	
Total	9 ± 4.301
Boy	8.333 ± 3.215
Girl	10 ± 7.071
**Gender (number, %)**	
Total	5 (100)
Boy	4 (80)
Girl	1 (20)
**Seizures type (number, %)**	
Total	5 (100)
Generalized tonic-clonic seizures	3(60)
Focal seizures	1(20)
Both	1 (20)
Others	0 (0)

**Table 4:** The detailed sequences of all primers used for RT-qPCR.

Name	Sequence
Human	
h-ZBP1 forward	5′-AACATGCAGCTACAATTCCAGA-3′
h-ZBP1 reverse	5′-AGTCTCGGTTCACATCTTTTGC-3′
h-GSDMD forward	5′-GTGTGTCAACCTGTCTATCAAGG-3′
h-GSDMD reverse	5′-CATGGCATCGTAGAAGTGGAAG-3′
h-RIPK1 forward	5′- GGGAAGGTGTCTCTGTGTTTC-3′
h-RIPK1 reverse	5′-CCTCGTTGTGCTCAATGCAG-3′
h-GAPDH forward:	5′- GGAGCGAGATCCCTCCAAAAT -3′
h-GAPDH reverse:	5′- GGCTGTTGTCATACTTCTCATGG -3′
**Mouse**	
*m*-ZBP1 forward:	5′-AAGAGTCCCCTGCGATTATTTG-3′
*m*-ZBP1 reverse:	5′-TCTGGATGGCGTTTGAATTGG-3′
*m*-GSDMD forward:	5′-ATGCCATCGGCCTTTGAGAAA-3′
*m*-GSDMD reverse:	5′-AGGCTGTCCACCGGAATGA-3′
*m*-RIPK1 forward:	5′-GACAGACCTAGACAGCGGAG-3′
*m*-RIPK1 reverse:	5′-CCAGTAGCTTCACCACTCGAC-3′
*m*-GAPDH forward:	5′- AGGTCGGTGTGAACGGATTTG -3′
*m*-GAPDH reverse:	5′- GGGGTCGTTGATGGCAACA -3′

### 2.13 Animals and drug treatment

To ascertain variations in the expression of ZBP1, GSDMD, and RIPK1 during childhood epileptogenesis, we utilized four-week-old C57BL/6J mice, following our previous research [9]. The design of this study's animal protocol was approved by the Animal Ethics Association of Jiangnan University, and all efforts were made to mitigate animal suffering and limit the number of animals used. Male C57BL/6J mice were procured from Gempharmatech Co., Ltd (Changzhou, China) and maintained under standard laboratory conditions, featuring a consistent temperature of 24 °C, a 12-h light/dark cycle, and unrestricted access to food and water. We categorized the mice into two groups randomly: 1) the KA group (n = 3), which received an intraperitoneal injection of 20 mg/kg kainic acid, and 2) the control group (n = 3), which administered an intraperitoneal injection of an equivalent volume of saline. One day after the KA injection, the mice were sacrificed, and their hippocampus was gathered for subsequent experiments.

### 2.14 Cell cultures

To verify the differences in ZBP1 expression, we used glutamate-induced excitotoxicity in HT22 cells as the experimental model of epilepsy in vitro, following the methodology described in our previous work [9]. Immortalized hippocampal neuronal cells (HT22) were obtained from Procell Life Science & Technology Co., Ltd. (CL-0697, Wuhan, China) and cultured in Dulbecco's Modified Eagle Medium (DMEM) (D5796, Sigma-Aldrich, St. Louis, USA) supplemented with 10% fetal bovine serum, 100 units of penicillin, and 100 μg/mL streptomycin. The cells were seeded in 6-well or 96-well plates and incubated at 37 °C in a 5% CO_2_ atmosphere for 24 h before the experiments. For the experiments, the cells were divided into two groups and treated for 24 h: 1) the control group; 2) the glutamate (Glu) group: cells were treated with 5 mM glutamate.

### 2.15 Western blot analysis

To quantify changes in protein expression levels in the samples, Western blot assays were conducted following the methodology described in our previous work [9]. Briefly, samples containing 10 μg of total protein were subjected to SDS-PAGE gel electrophoresis to separate the proteins based on their molecular weight. The separated proteins were subsequently transferred onto PVDF membranes. The membrane was then blocked using TBST buffer supplemented with 5% nonfat milk for 1 h at room temperature. Following blocking, the membrane was incubated overnight at 4 °C with primary antibodies specific to ZBP1, and β-actin protein (obtained from Proteintech Group, Inc, Shanghai, China). Secondary antibodies (obtained from Cell Signaling Technology, MA, USA) were diluted in 5% nonfat milk in TBST and added to the membrane for 1 h at room temperature.

### 2.16 Quantitative real-time reverse transcription PCR (qRT-PCR)

In this study, qRT-PCR was employed to assess the mRNA level of ZBP1, GSDMD, and RIPK1 in the hippocampus or HT22 cells, following the methodology outlined in our previous work [9]. Briefly, total RNA was extracted from the hippocampus or HT22 cells using RNAzol reagent. The extracted RNA was then reverse transcribed into complementary DNA (cDNA) using a Takara kit from Shiga, Japan. The relative expression of the target gene was normalized to the glyceraldehyde 3-phosphate dehydrogenase (GAPDH) gene and analyzed using the 2^(-ΔΔCt) method.

### 2.17 Statistical analysis

The statistical analyses were performed using the R software. Continuous variables were presented as the standard error of the mean (SEM). The normal distribution of the data was assessed using the Shapiro-Wilk test. For normally distributed data, the Student's t-test or the Wilcoxon rank sum test was used for comparisons. For non-normally distributed data, the chi-square test was employed for comparisons of categorical data. In this study, statistical significance was defined as a p-value less than 0.05. The results were reported with *P < 0.05 and **P < 0.01 to indicate the significance level.

## 3 Results

### 3.1 Analysis of time-course expression patterns of PANoptosis-related genes in KA-induced epileptic mice

In the study, differential expression analysis was performed on the GSE99577 dataset using the DESeq2 R package. Initially, a total of 16,172 differentially expressed genes (DEGs) were detected at six different time points after KA injection. Then, using adjacent time points as controls, 12,879 DEGs were identified. Additionally, the ImpulseDE2 R package was utilized to further screen for DEGs related to KA-induced epilepsy, resulting in the identification of 21,074 DEGs. In total, 21,500 DEGs were obtained, as depicted in Fig. 2B of the study. These DEGs are likely to play a significant role in the context of KA-induced epilepsy.

To investigate the dynamic changes of the 21,500 differentially expressed genes (DEGs) over time, a time series analysis was performed. The optimal number of clusters for grouping the DEGs was 5, as depicted in Fig. 2C. The line graph on the left side of Fig. 2D illustrates the distinct time expression patterns exhibited by the DEGs within each of the 5 clusters. Among the clusters identified, Cluster 5 (n = 5453) demonstrated a decreasing trend in gene expression levels before reaching a low point at 1 day, followed by an increasing trend. Conversely, Cluster 2 (n = 5304) exhibited the opposite pattern, with gene expression levels showing an initial increase followed by a decrease. In Cluster 4 (n = 4851), gene expression levels increased immediately after KA injection. Cluster 3 (n = 3405) displayed gene expression levels that peaked at 1 day. Finally, in Cluster 1 (n = 2487), gene expression levels began to decline at 2 days. These distinct expression patterns within each cluster provide valuable insights into the dynamic changes of gene expression over time.

The biological processes and pathways associated with each time cluster were further investigated through GO and KEGG analyses. As depicted in Fig. 2D, the genes in Cluster 5 were primarily associated with the biological process of synapse organization, with a notable enrichment in the Retrograde endocannabinoid signaling pathway. In contrast, the genes in Cluster 2 were predominantly linked to the biological process of DNA-templated, particularly in the DNA replication pathway. The expression of genes in Cluster 4 exhibited a strong association with the biological process of lymphocyte differentiation, with a significant focus on the Coronavirus disease-COVID-19 pathway. Cluster 3 showed a high correlation with the biological process of positive regulation of response to external stimulus, mainly concentrated in the Neuroactive ligand-receptor interaction pathway. Lastly, the genes in Cluster 1 displayed a significant relationship with the biological process of ribonucleoprotein complex biogenesis, primarily enriched in the Proteasome pathway. These findings shed light on the specific biological processes and pathways associated with each time cluster, providing valuable insights into the functional implications of the gene expression patterns observed in this study.

### 3.2 PANoptosis-related genes can distinguish between the control and KA-induced epileptic mice

**Fig. 3:**
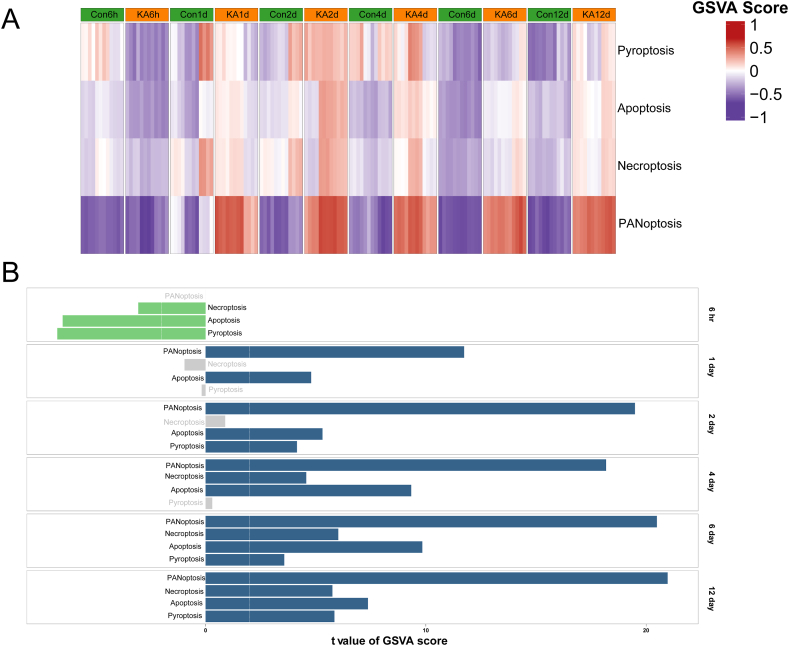
GSVA analysis shows that PANoptosis Gene Set can distinguish Con and KA in vivo model. (A) Heatmap of Apoptosis, Pyroptosis, Necroptosis, and PANoptosis gene set expression between con and KA at different time points. (B) Results of differential analysis of GSVA Score. The white dashed lines represent the threshold of significant difference; green rectangle indicates a significant downregulation of the pathway; blue rectangle indicates a significant upregulation of the pathway, and grey rectangle indicates no significant difference in the pathway. (For interpretation of the references to colour in this figure legend, the reader is referred to the Web version of this article.)

To systematically investigate the role of PANoptosis-related genes in KA-induced epileptic mice, GSVA was conducted using gene lists associated with PANoptosis, Apoptosis, Pyroptosis, and Necroptosis. The GSVA Score heatmap, as depicted in Fig. 3A and Table 3, demonstrated that only the PANoptosis genes set can effectively distinguish between the control and KA groups at the five different time points (1, 2, 4, 6, and 12 days). Additionally, the differential analysis of GSVA scores revealed that the PANoptosis gene list exhibited a significant upregulation in pathway activity from 1 day to 12 days, as illustrated in Fig. 3B. These findings suggest that PANoptosis-related genes play a prominent role in the KA-induced epileptic mice model and highlight the importance of PANoptosis in the progression of epilepsy.

**Table 3:** Expression of PANoptosis genes at different time points.

PANoptosis genes	6hr	1 day	2 day	4 day	6 day	12 day
STAT1	Stable	Stable	Up	Up	Up	Up
INOS(NOS2)	Down	Up	Up	Up	Up	Up
MAP3K7(TAK1)	Stable	Stable	Stable	Stable	Stable	Stable
TNFR1(Tnfrsf1a)	Stable	Up	Up	Up	Up	Up
IRF1	Down	Stable	Up	Up	Up	Up
TNF	Stable	Stable	Up	Up	Up	Up
IFNG(IFNs)	Stable	Stable	Up	Stable	Up	Up
ZBP1(DAI/DLM1)	Stable	Up	Up	Up	Up	Up
AIM2	Stable	Up	Up	Up	Up	Up
RIPK1	Stable	Up	Up	Up	Up	Up
RIPK3	Stable	Up	Up	Up	Up	Up
NLRP3	Stable	Up	Up	Up	Up	Up
CASP8	Up	Up	Up	Up	Up	Up
FADD	Stable	Up	Up	Up	Up	Up
CASP1	Stable	Up	Up	Up	Up	Up
PYCARD (ASC)	Stable	Up	Up	Up	Up	Up
MEFV(Pyrin)	Stable	Up	Up	Up	Up	Up
CASP6	Stable	Up	Up	Up	Up	Up
IL18	Stable	Up	Up	Up	Up	Up
IL1B	Stable	Up	Up	Up	Up	Up
CASP3	Stable	Up	Up	Up	Up	Up
CASP7	Down	Up	Up	Up	Up	Up
GSDME	Stable	Down	Down	Down	Down	Down
GSDMD	Stable	Up	Up	Up	Up	Up
MLKL	Stable	Up	Up	Up	Up	Up

### 3.3 These PANoptosis-related biomarkers are associated with neuron loss based on WGCNA analysis

**Fig. 4:**
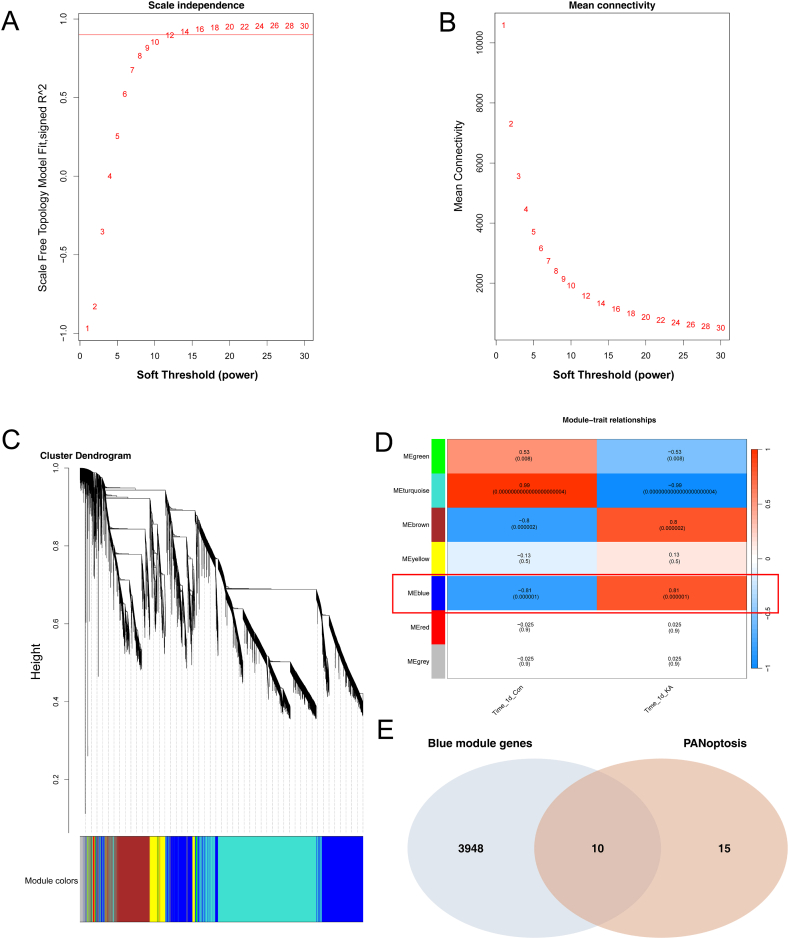
10 PANoptosis biomarkers associated with neuronal damage identified by WGCNA. (A) The scale-free fit index analysis. (B) The mean connectivity analysis. (C) WGCNA divides all genes into 7 different gene modules and clustering results. (D) Correlation of each gene module with KA. The first line of each cell shows the correlation coefficient, and the second line displays the P-value. (E) The intersection of Blue module genes and PANoptosis-related genes. (For interpretation of the references to colour in this figure legend, the reader is referred to the Web version of this article.)

In the KA-induced epileptic mice model, the different phases of epileptic progression are defined as follows: the acute phase encompasses the first day (0–24 h) after KA stimulation, the latent phase spans from 2 to 14 days, and the chronic phase occurs after 14 days [31]. To identify PANoptosis-related biomarkers associated with neuronal damage in this model, a WGCNA analysis was conducted. Using a soft threshold of 12 (R-Squared = 0.91, Fig. 4A and B), the analysis identified 7 distinct gene modules. Among these modules, the blue gene module (n = 5721) demonstrated the strongest association with the occurrence and development of epilepsy, as depicted in Fig. 4C. To identify hub genes within the blue module, the KME method was employed. A total of 3958 hub genes were identified, and an intersection was performed with the PANoptosis-related biomarkers. This intersection revealed several marker genes that are highly relevant to PANoptosis in the mechanism of neuronal damage following an epileptic seizure. Remarkably, the intersection identified 10 marker genes: MLKL, IRF1, RIPK1, GSDMD, CASP1, CASP8, ZBP1, CASP6, PYCARD, and IL-18 (Fig. 4D). These genes are suggested to be the pivotal molecular components involved in PANoptosis, and could be critical in the underlying mechanisms of neuronal damage following epileptic seizures.

### 3.4 The PANoptosis-related genes are potential diagnostic biomarkers of epilepsy

**Fig. 5:**
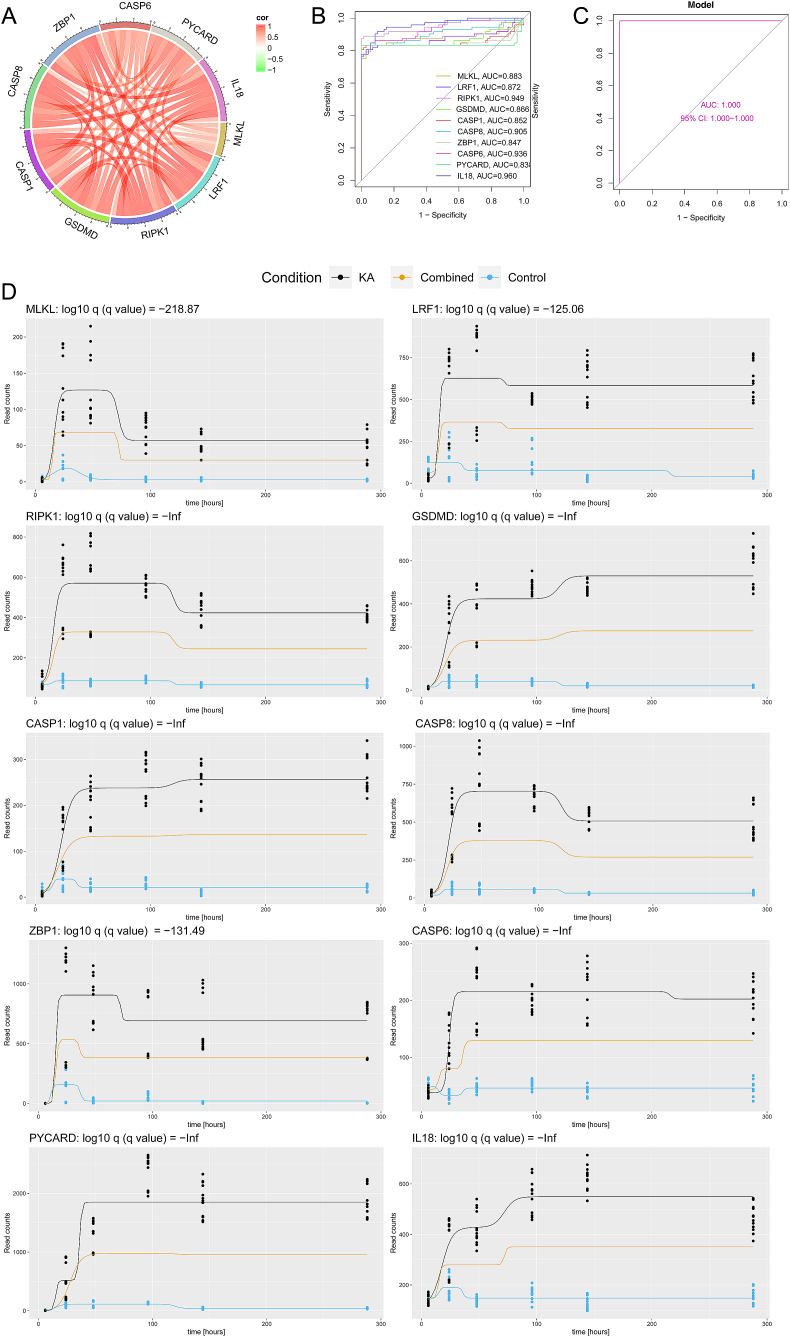
Correlation analysis of marker genes and disease diagnosis model. (A) ROC curves and for the 10 marker genes in GSE99577. (B) Logistic regression model to identify the AUC of disease samples in GSE99577. (C) Correlations among the 10 marker genes. (D) The time expression mode of 10 PANoptosis marker genes. The black lines represent gene expression in the hippocampus of KA-induced epileptic mice, blue lines represent gene expression in the hippocampus of control mice, and orange lines represent combined gene expression in the hippocampus of both KA and control groups. The x-axis represents time points in hours, and the y-axis represents read counts of genes. (For interpretation of the references to colour in this figure legend, the reader is referred to the Web version of this article.)

Following the identification of 10 PANoptosis-related biomarkers through WGCNA analysis, a correlation analysis was conducted among these genes, revealing strong correlations among them, as depicted in Fig. 5A. To assess the diagnostic potential of these 10 biomarkers in epileptic mice, their performance was evaluated at different time points following KA or NaCl injection. The results, shown in Fig. 5B and C, indicated that all 10 marker genes displayed high accuracy and predictive capability, as their AUC (Area Under the Curve) scores surpassed 0.7. This suggests that these markers could serve as potential indicators of epilepsy. Additionally, Fig. 5D illustrates the time expression patterns of the 10 PANoptosis-related genes are associated with neuron loss. These patterns provide valuable insights into the dynamic changes in gene expression during the progression of epilepsy. Furthermore, the time expression mode of 10 PANoptosis marker genes associated with neuron loss is shown in Fig. 5D. Taken together, these findings underscore the potential diagnostic significance of the 10 marker genes as a collective panel in accurately diagnosing epilepsy.

### 3.5 GSEA prediction of possible participation pathways of PANoptosis marker genes

**Fig. 6:**
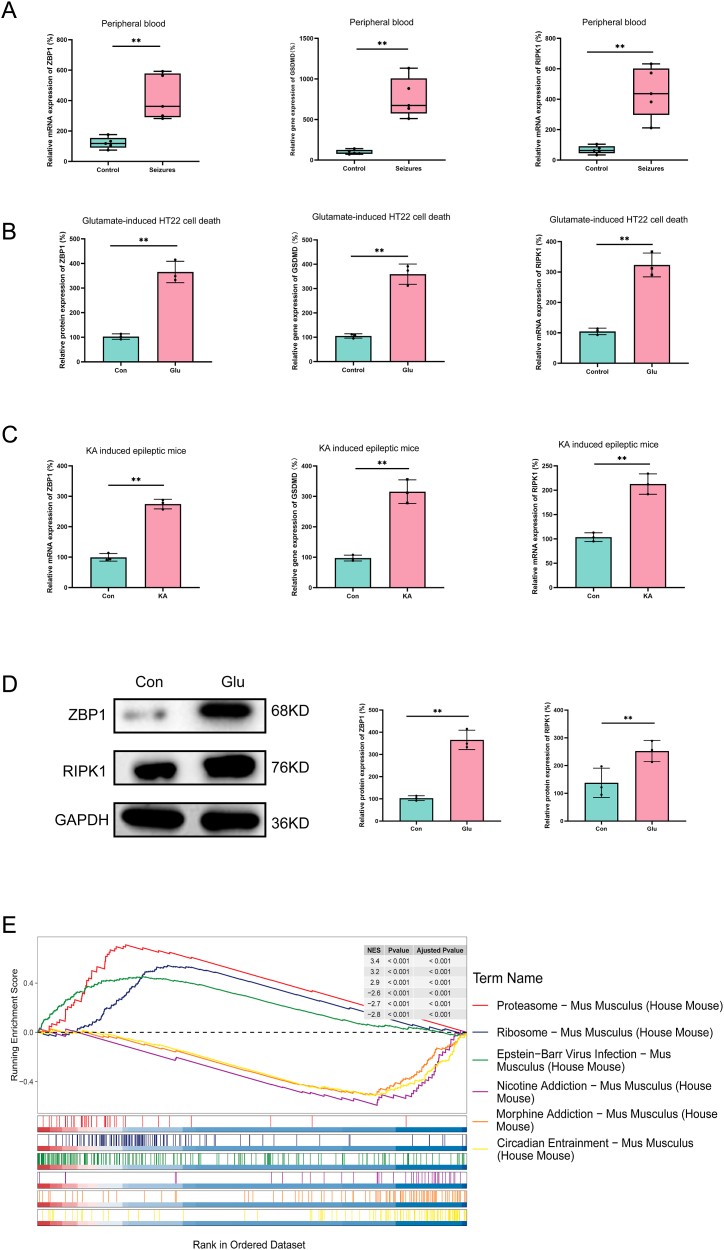
Experiments validation and ssGSEA prediction of possible participation pathways of PANoptosis marker genes. (A) mRNA expression of ZBP1,GSDMD, and RIPK1 in peripheral blood between healthy school-aged children and age-matched, newly diagnosed, untreated patients with seizures. (B) mRNA expression of ZBP1,GSDMD, and RIPK1 in glutamate induced cell death model. (C) mRNA expression of ZBP1,GSDMD, and RIPK1 in kainic acid-induced epileptic mice model. (D) protein expression of ZBP1 in glutamate induced cell death model. (E) ssGSEA prediction of possible participation pathways of PANoptosis marker genes.

To understand the biological pathways in which PANoptosis marker genes partake, we applied a single-gene gene set enrichment analysis (ssGSEA) on the mentioned 10 marker genes. We also conducted various experiments to validate the expression changes of three PANoptosis markers (ZBP1, GSDMD, and RIPK1) during the pathophysiological process of epilepsy. As shown in Fig. 6A, we observed a significant increase in the mRNA expression of ZBP1, GSDMD, and RIPK1 in the peripheral blood of newly diagnosed seizure patients compared to age-matched healthy children (P < 0.01), suggesting their involvement in epilepsy onset. Additionally, we assessed the mRNA of ZBP1, GSDMD, and RIPK1 and the protein expression of ZBP1 and RIPK1 in epilepsy models in vitro. Fig. 6B–C shows a significant increase in ZBP1, GSDMD, and RIPK1 expression compared with the control group (P < 0.01), which confirms their expression change during epilepsy occurrence, and supports their potential involvement in epilepsy pathogenesis. Moreover, the ssGSEA results indicated the potential involvement of the 10 marker genes in various signaling pathways. The top 3 upregulated pathways associated with these genes were Proteasome, Ribosome, and Epstein-Barr Virus infection. Conversely, the top 3 downregulated pathways were Nicotine addiction, Morphine addiction, and Circadian entrainment signaling pathways (Fig. 6D). Collectively, these findings suggest that PANoptosis may play a role in these pathways during the development and progression of epilepsy.

## 4 Discussion

Epilepsy, a common chronic neurological condition in children, is characterized by hippocampal neuronal cell death, which plays a significant role in the underlying pathophysiological processes of seizures [32,33]. In recent years, PANoptosis has emerged as a novel form of cell death that contributes to various neurological disorders, such as Alzheimer's disease, Parkinson's disease, and amyotrophic lateral sclerosis [12]. PANoptosis involves the simultaneous activation of pyroptosis, apoptosis, and necroptosis within the same cell population and is regulated by the PANoptosome, a cytoplasmic protein complex [13]. However, the specific role of PANoptosis in the context of seizures is not yet fully understood. Therefore, this study aimed to investigate the involvement of PANoptosis in epilepsy using an integrated approach combining bioinformatics analysis and experimental validation. The overall goal of this study is to provide valuable insights into the role of PANoptosis in epilepsy and its potential implications for the development of targeted therapeutic approaches.

The direct evidence of PANoptosis involvement in neurological disorders is currently limited, mainly derived from in vitro experiments or animal models [12]. Our study sought to provide evidence that PANoptosis is implicated in the pathophysiological processes of seizures, which is a significant step toward understanding the molecular mechanisms underlying epilepsy. To achieve this, we conducted a time series analysis to examine the temporal expression patterns of PANoptosis-related genes. Our results revealed dynamic changes in these genes expression, particularly at 1 day following seizure induction. The GSVA further demonstrated that PANoptosis-related genes effectively distinguished between control and KA-induced epileptic groups at different time points. This emphasized the importance of PANoptosis in epilepsy progression and demonstrated the significant role these genes played in the KA-induced epileptic mice model. Moreover, the weighted Gene co-expression network analysis (WGCNA) revealed a strong association between PANoptosis-related genes and the occurrence and development of epilepsy. We identified biomarker genes (including MLKL, IRF1, RIPK1, GSDMD, CASP1, CASP8, ZBP1, CASP6, PYCARD, and IL18) that exhibited high diagnostic accuracy and predictive ability for epilepsy. There is increasing evidence suggesting that peripheral blood gene expression can reflect molecular changes occurring in the brain or other tissues affected by neurological diseases [34,35]. In this study, we initially validated our bioinformatics findings by comparing the gene expression differences of ZBP1, GSDMD, and RIPK1. We observed a significant increase in the expression of ZBP1, GSDMD, and RIPK1 in newly diagnosed, untreated patients with seizures compared to age-matched healthy children (P < 0.01). Previous studies have shown that elevated levels of glutamate in the brain tissues of patients with temporal lobe epilepsy, and it has been demonstrated that glutamate-induced excitotoxicity causes neuronal death in epilepsy [36,37]. Moreover, blocking glutamate receptors can reduce seizure frequency and severity [38]. Therefore, glutamate-induced cell death and kainic-acid epileptic mice were often used as the experimental model of epilepsy in vitro and in vivo, respectively [8,9]. In this study, we then assessed the mRNA of ZBP1, GSDMD, and RIPK1 and the protein expression of ZBP1 and RIPK1 in epilepsy models in vitro. Similar to the results of gene expression in peripheral blood, there is a significant increase in the mRNA expression of ZBP1, GSDMD, and RIPK1 compared to the control group (P < 0.01). There is also a significant increase in the protein expression of ZBP1 and RIPK1 compared to the control group (P < 0.01). Our results confirmed changes in ZBP1, GSDMD, and RIPK1 expression during epilepsy onset, further supporting the potential involvement of PANoptosis in seizure pathogenesis. In summary, our findings offer a deeper understanding of epilepsy's molecular mechanisms, aiding in early detection, management, and potentially improving diagnostic accuracy. Importantly, our results may help identify novel therapeutic targets for epilepsy, presenting new avenues for future therapeutic interventions.

Programmed cell death (PCD) is a process in which cells die in a controlled and regulated way [39]. Previous studies indicated that PCD plays a critical role in the development of the nervous system and provided evidence supporting the involvement of PCD, such as apoptosis, necroptosis, and pyroptosis, in the pathophysiological processes of seizures [7,40,41]. However, the precise mechanisms underlying the crosstalk between these cell death pathways in the context of seizures have yet to be fully elucidated. Oxidative stress is a condition in which the balance between reactive oxygen species (ROS) and antioxidants is disturbed, leading to cellular damage. Previous studies demonstrated that oxidative stress can trigger or modulate the apoptosis, pyroptosis, ferroptosis, and necroptosis [39,42]. In recent years, emerging evidence indicated a newly formed PCD called PANoptosis is closely related to oxidative stress [43]. Oxidative stress can induce or promote PANoptosis, thereby contributing to the occurrence and development of various diseases, including cancer, neurodegenerative disorders, and ischemia-reperfusion injury. Conversely, PANoptosis can also lead to the generation and release of ROS, thereby exacerbating oxidative stress and triggering extracellular inflammation [19,44]. Interestingly, it has been suggested that another form of PCD, ferroptosis, which is implicated in several neurological diseases such as cerebral ischemia, Alzheimer's disease, and Parkinson's disease, is closely linked with PANoptosis [45]. The link between ferroptosis and PANoptosis might be attributed to mitochondrial dysfunctions, which can induce oxidative stress and iron dyshomeostasis, thereby activating both ferroptosis and PANoptosis. However, the exact mechanism between oxidative stress and PANoptosis in seizures and their potential contributions to the pathogenesis of epilepsy remain areas of active investigation [46]. Understanding the intricate interactions between these processes may provide valuable insights into the underlying mechanisms and offer potential therapeutic targets for managing seizures and associated neurological disorders. Further research is needed to elucidate the precise molecular mechanisms linking oxidative stress, PANoptosis, and seizures. The findings of our study provide valuable insights into the potential development of novel therapeutic approaches for epilepsy, shedding light on the exploration of PANoptosis as a target for epilepsy treatment [47].

However, this study has several limitations that should be considered. First, The KA model in mice used in this study is a widely used experimental for temporal lobe epilepsy, the most common type of partial epilepsy in adults. However, pediatric epilepsy often arises from genetic factors and developmental disorders, differing significantly from adult epilepsy, which is usually triggered by trauma, infections, or degenerative diseases. Therefore, the findings from this study may not be directly applicable to pediatric epilepsy. Future research should investigate the role of the PANoptosis pathway in pediatric epilepsy. Secondly, the connections between epilepsy and specific forms of PANoptosis, namely pyroptosis and necroptosis, remain inadequately explored. There is a need for direct evidence demonstrating the crosstalk between necroptosis and pyroptosis, and the crosstalk between necroptosis and apoptosis in the context of epilepsy. Moreover, the clinical value of PANoptosis-related biomarkers should be further elucidated using clinical data. Finally, the underlying mechanism by which PANoptosis contributes to seizures is largely unknown. Additional in vitro and in vivo studies are needed to deepen our understanding of these complex processes.

## 5 Conclusion

To the best of our knowledge, our study is the first to propose a potential association between hippocampal neuronal cell death in epilepsy and PANoptosis, a newly identified form of cell death. This novel finding provides valuable insights into the underlying pathophysiological processes involved in epilepsy. These findings may contribute to the development of novel therapeutic targets and the interventions aimed at modulating PANoptosis to mitigate hippocampal neuronal cell death and improve outcomes for individuals with epilepsy.

## Data availability statement

Data are available on request to corresponding authors via emailing.

## Additional information

This work was supported by the Research project of the 10.13039/501100016308Wuxi Health and Family Planning Commission (MS201760, to Dr.Cao). Top Talent Support Program of Wuxi Health Committee (No: BJ2020042, to Dr. Liu) and Wuxi Key Medical Talents financial (No: ZDRC017, to Dr. Liu). Postgraduate Research & Practice Innovation Program of Jiangsu Province (No: KYCX22_2434, to Dr. Xie).

## CRediT authorship contribution statement

**Yueying Liu:** Writing – original draft, Validation, Formal analysis, Data curation, Conceptualization. **Yuanjin Chang:** Writing – original draft, Validation, Formal analysis, Data curation, Conceptualization. **Xiaofan Jiang:** Writing – original draft, Validation, Formal analysis, Data curation, Conceptualization. **Huiya Mei:** Writing – original draft, Visualization, Validation, Methodology. **Yingsi Cao:** Writing – review & editing, Writing – original draft, Formal analysis, Data curation. **Dongqin Wu:** Writing – review & editing, Writing – original draft, Formal analysis, Data curation. **Ruijin Xie:** Writing – review & editing, Software, Methodology. **Wenjun Jiang:** Writing – review & editing, Software, Methodology. **Emely Vasquez:** Writing – review & editing, Software, Methodology. **Yu Wu:** Writing – review & editing, Supervision, Funding acquisition. **Shunyan Lin:** Writing – review & editing, Supervision, Funding acquisition. **Yachuan Cao:** Writing – review & editing, Supervision, Funding acquisition.

## Declaration of competing interest

The authors declare that they have no known competing financial interests or personal relationships that could have appeared to influence the work reported in this paper.
