# Supplementary figures and images for: Analysis of the role of PANoptosis in seizures via integrated bioinformatics analysis and experimental validation
Source: Heliyon. 2024 Feb 14;10(4):e26219. doi: 10.1016/j.heliyon.2024.e26219 (PMC10884430; doi:10.1016/j.heliyon.2024.e26219)

Con Glu

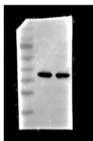

Repeat1

Con Glu

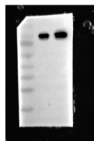

Con Glu

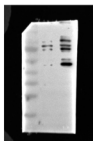

GAPDH 36Kd

RIPK1 76Kd

ZBP1 46Kd

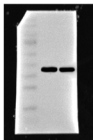

Repeat2

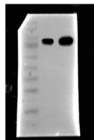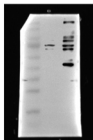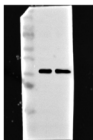

Repeat3

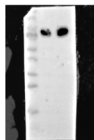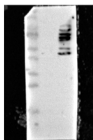

Supplement: Multimedia component 1 [file mmc1.pdf]
